# Diamond formation in the deep lower mantle: a high-pressure reaction of MgCO_3_ and SiO_2_

**DOI:** 10.1038/srep40602

**Published:** 2017-01-13

**Authors:** Fumiya Maeda, Eiji Ohtani, Seiji Kamada, Tatsuya Sakamaki, Naohisa Hirao, Yasuo Ohishi

**Affiliations:** 1 Department of Earth Science, Graduate School of Science, Tohoku University, Sendai, 980-8578, Japan; 2 V.S. Sobolev Institute of Geology and Mineralogy, SB RAS, Novosibirsk, 630090, Russia; 3 Frontier Research Institute for Interdisciplinary Sciences, Tohoku University, Sendai, 980-8578, Japan; 4 Japan Synchrotron Radiation Research Institute, Sayo, Hyogo 679-5198, Japan

## Abstract

Diamond is an evidence for carbon existing in the deep Earth. Some diamonds are considered to have originated at various depth ranges from the mantle transition zone to the lower mantle. These diamonds are expected to carry significant information about the deep Earth. Here, we determined the phase relations in the MgCO_3_-SiO_2_ system up to 152 GPa and 3,100 K using a double sided laser-heated diamond anvil cell combined with *in situ* synchrotron X-ray diffraction. MgCO_3_ transforms from magnesite to the high-pressure polymorph of MgCO_3_, phase II, above 80 GPa. A reaction between MgCO_3_ phase II and SiO_2_ (CaCl_2_-type SiO_2_ or seifertite) to form diamond and MgSiO_3_ (bridgmanite or post-perovsktite) was identified in the deep lower mantle conditions. These observations suggested that the reaction of the MgCO_3_ phase II with SiO_2_ causes formation of super-deep diamond in cold slabs descending into the deep lower mantle.

Carbon is circulated around the surface and interior of the Earth with subducting slabs and volcanic eruptions; subduction carries carbon-bearing rocks to the Earth’s interior and volcanic eruption expels carbon-bearing gas, lavas and rocks from the interior of the Earth^1^. The flux of subducted carbon within oceanic plates is estimated to be more than 5 Tmol/yr, almost twice as large as the expelled-carbon flux, 2–3 Tmol/yr, through arc magmatism^2^. This difference suggests the existence of carbon reservoirs in the deep Earth^1^,^2^.

One source of direct evidence for deep carbon is carbon-bearing samples originating from the Earth’s interior. Diamond is evidence of quite a deeper-origin carbon. In particular, some diamonds, called ‘super-deep diamond’, are thought to arise from the mantle transition zone or the lower mantle^3^,^4^,^5^,^6^,^7^,^8^. The inclusions in super-deep diamond may supply information on the lithology, water content, and/or elemental distribution in deep parts of the Earth^3^,^4^,^5^,^6^,^7^,^8^.

Subducting slabs play a key role for carrying carbon-bearing phases into the deep Earth and forming super-deep diamond^3^,^4^,^5^. Altered rocks in the oceanic crust contains the large amount of carbon such as organic carbon or carbonate minerals, which can be the deep reservoir of subducted carbon^9^,^10^. These carbonate minerals or melts may change to diamond if they become unstable in the Earth during the subduction process^3^,^5^,^11^,^12^,^13^. The stability of carbonates has been investigated using high-pressure experiments and *ab initio* calculations, and MgCO_3_ magnesite is determined to be stable under the high-pressure and high-temperature conditions expected during subduction^11^,^12^,^13^,^14^,^15^,^16^,^17^,^18^,^19^,^20^. The existence of carbonate (or carbonatite melt) in the deep mantle is supported by the discovery of carbonate as inclusions in diamonds originating from the mantle transition zone and/or the lower mantle^3^,^5^.

Since SiO_2_ is one of the abundant components and also is an important phase in deeply subducted slabs^21^,^22^,^23^, the MgCO_3_-SiO_2_ system may be applied to the slabs descending into the lower mantle. SiO_2_ phases may change to its high-pressure polymorph, such as coesite, stishovite, CaCl_2_-type phase and seifertite^24^,^25^. Magnesite is expected to break down to CO_2_ or diamond by reacting with silica minerals in the MgCO_3_-SiO_2_ system in subducting process^12^,^13^,^26^,^27^ although the detail of its phase relation has not yet been clarified. Knowledge of the reactions in this system at high pressure and high temperature may provide important insights into the carbon-related processes in the deep mantle, such as the origin of super-deep diamond and melting or oxidation by release of volatile components^13^,^28^. We used a laser-heated diamond anvil cell (LHDAC) combined with a high-pressure and high-temperature *in situ* synchrotron X-ray diffraction (XRD) technique to quantify the phase relations of the MgCO_3_-SiO_2_ system down to the lowermost-mantle conditions. Our objective is to clarify the behavior of carbon in the lower mantle and to model the origin of super-deep diamond.

## Results

We observed the phase relation of MgCO_3_ and SiO_2_ system up to 152 GPa and 3,100 K (see Supplementary Tables S1 and S3). Diamond and bridgmanite may be formed through the following reactions^12^,^13^,^28^:


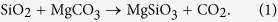



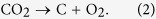


Or, the following reaction is also possible:


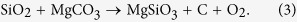


**Figure 1:**
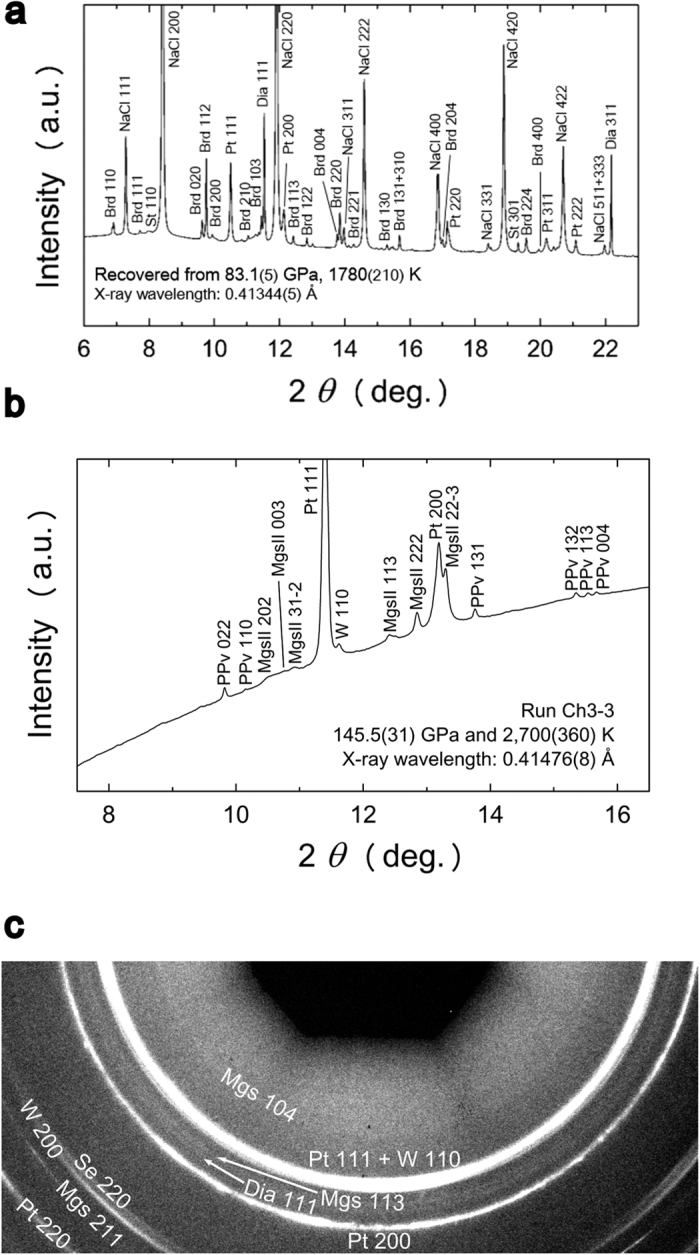
XRD patterns of the reaction products in the MgCO_3_-SiO_2_ system. (**a**) XRD patterns obtained under the ambient condition for the samples recovered from 83.1(5) GPa and 1,780(210) K. The X-ray pattern was taken at ambient condition without DAC, of which surface showed no damage after the experiment. The abbreviations represent as follow: St: stishovite, Brd: bridgmanite, Dia: diamond, Pt: platinum, W: tungsten (gasket), NaCl: sodium chloride (pressure medium). (**b**) *In situ* XRD patterns obtained at 145.5 (31) GPa and 2,700 (360) K. (**c**) 2D XRD images of a sample recovered from 145.5 (31) GPa and 2,700 (360) K obtained at ambient condition without DAC. The abbreviations represent as follow: Se: seifertite, Mgs: magnesite, MgsII: MgCO_3_ phase II, PPv: MgSiO_3_ post-perovskite phase, Dia: diamond, Pt: platinum, W: tungsten (gasket).

Bridgmanite and diamond were observed using XRD, in a run product recovered from 83 GPa and 1,780 K (Fig. 1a). The X-ray pattern was taken at an ambient condition without DAC, of which surface showed no damage after the experiments. The high-pressure phase of CO_2_ was not detected in most runs, matching the previous studies on the MgCO_3_-SiO_2_ system^12^,^13^. We successfully detected the high-pressure phase of CO_2_, CO_2_-VI^29^ in one run made by *in situ* X-ray diffraction at 83 GPa and 1,780 K (see Supplementary Table S1). Thus, occurrence of reactions (1) and (2) or (3) were thus confirmed.

The structures of the MgCO_3_ high-pressure phases and their phase transition boundary are controversial and the data on their compression behaviors are very limited. A recent *ab initio* study reported a monoclinic post-magnesite phase, MgCO_3_ phase II (C2/m) at 300 K and pressures from 82 to 138 GPa^16^. On the other hand, the latest study reported the stabilization of another post-magnesite phase, having space group P-1, at 300 K and 85–101 GPa, which transforms to MgCO_3_ phase II (C2/m) at 101 GPa and 300 K^19^. We conducted an *in situ* XRD study of MgCO_3_ using a double sided laser-heated diamond anvil cell in the pressure range from 85 GPa to 132 GPa at about 2,500–3,000 K. Diffraction peaks after heating corresponded to the MgCO_3_ phase II^16^ in the same pressure range (see Supplementary Fig. S1 and Supplementary Table S2). The volumes of MgCO_3_ phase II in each run were estimated by fitting XRD patterns (see Supplementary Table S2). We then fitted the estimated volumes using the second order Birch-Murnaghan equations of state (BMEOS). As a result, the unit-cell volume at ambient condition (V_0_) and isothermal bulk modulus (K_300K, 0_) of magnesite phase II were estimated to be 498.9(5) Å^3^ and 154.9(7) GPa, respectively (K’ = 4; fixed). These values are consistent with the V_0_ and K_T0_ calculated by ref. 16, V_0_ = 503.36 Å^3^ (Z = 12) and K_0_ = 156.76 GPa using the third order BMEOS (K’ = 4.12).

MgCO_3_ phase II was identified in MgCO_3_-SiO_2_ system at pressures above 100 GPa by comparing the XRD patterns of the runs in the MgCO_3_-SiO_2_ system with the results of MgCO_3_ compression (see Supplementary Information Table S2). This enabled us to distinguish the MgSiO_3_ bridgmanite/post-perovskite phase from MgCO_3_ phase II in the XRD patterns (Fig. 1b). The MgSiO_3_ post-perovskite phase is thought to be formed by either reaction (1) or (3), where MgCO_3_ and SiO_2_ are considered to be MgCO_3_ phase II and seifertite, respectively. XRD spots (111) indicating diamond were also observed in some runs conducted at pressures greater than 100 GPa (see Supplementary Fig. S2). The number of diamond spots was limited and their intensities were weak. They did not appear in all 1D XRD patterns at high pressure. However, diamond created in high-pressure and high-temperature conditions was confirmed in the recovered run products (Fig. 1a and c).

**Figure 2:**
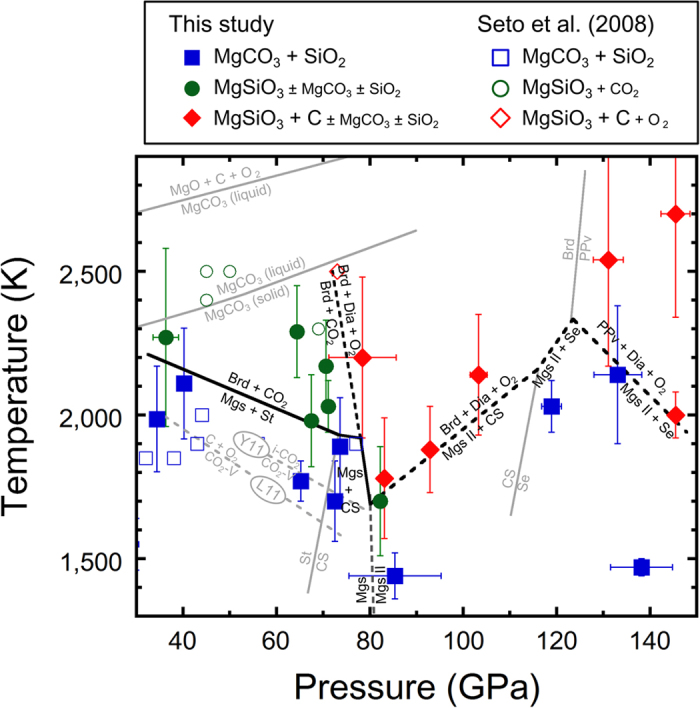
Phase diagram of the MgCO_3_-SiO_2_ system. The closed and open symbols represent the results of the present study and ref. 13, respectively. The colors of the symbols show the observed phases as follow: blue: MgCO_3_ + SiO_2_, green: MgSiO_3_ ± MgCO_3_ ± SiO_2_ (+CO_2_), red: MgSiO_3_ + C ± MgCO_3_ ± SiO_2_. The present phase boundaries in the MgCO_3_-SiO_2_ system are shown by black solid and dotted lines. The non-equilibrium phases are shown as a small font in the index column above the phase diagram. The referred phase boundaries between CO_2_-V and ionic CO_2_^30^ and between CO_2_-V and C + O_2_^28^ are gray dotted lines, shown by ‘Y11’ and ‘L11’, respectively. The phase boundaries of SiO_2_ stishovite (St)-to-CaCl_2_-type SiO_2_ (CS)^24^ and CaCl_2_-type SiO_2_ (CS)-to-seifertite (Se)^25^, the phase boundary of MgSiO_3_ bridgmanite (Br)-to-post-perovskite phase (PPv)^37^, and those of MgCO_3_ magnesite-to-MgCO_3_ liquid and MgCO_3_ liquid-to-MgO + CO_2_^20^ are shown by gray solid lines.

Figure 2 shows the phase diagram of the MgCO_3_-SiO_2_ system based on the present and previous studies. The temperature of magnesite decarbonation is consistent with ref. 13 but higher than ref. 12 up to 70 GPa. We discovered the phase transformations from CaCl_2_-type SiO_2_ + MgCO_3_ phase II to bridgmanite + diamond + O_2_ and from seifertite + MgCO_3_ phase II to bridgmanite/post-perovskite + diamond + O_2_ for the first time in this experiment. The decomposition boundary of CO_2_ has a steep gradient in the present phase diagram of MgCO_3_-SiO_2_ system which is not consistent with the results of any previous studies on CO_2_, such as decomposition^28^ or phase transition of CO_2_^30^.

## Discussion

**Figure 3:**
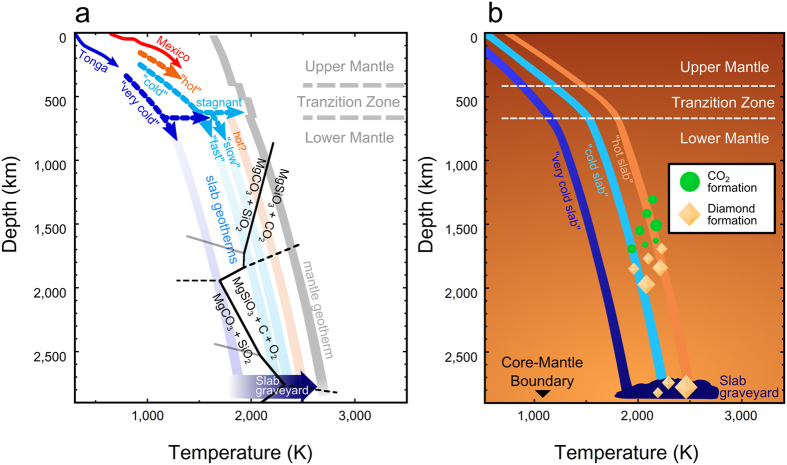
Change of carbon-bearing phases in the slabs descending into the lower mantle. (**a**) The phase boundaries in the MgCO_3_-SiO_2_ system are denoted with the Earth’s geotherms. The gray solid zone shows the mantle adiabat^33^. The blue and red solid arrows are the depth-temperature paths of Tonga and Mexico subduction zones, where the coldest and hottest slabs were modeled in ref. 32, respectively. The hypothetical models of the “hot”, “cold” and “very cold” slabs^31^ are shown by the orange, light blue and blue arrows, respectively, and the zone of each color is extrapolation of each path. The series of paths of cold-slab geotherm below the mantle transition zone represent the case of the fast subduction, stagnant and the slow subduction^31^. (**b**) The schematic model of the super-deep diamond’s formation. The orange, light blue and blue lines represent the “hot”, “cold” and “very cold” subducting slabs^31^, respectively. The green circles and diamond-shaped symbols indicate the possible depth of CO_2_ and diamond formation, respectively.

Since the reactions between MgCO_3_ and SiO_2_ may be expected in deeply subducted slabs^13^,^26^,^27^, we should consider the phase relations in the MgCO_3_-SiO_2_ system on subducting depth-temperature paths. The phase boundaries of the MgCO_3_-SiO_2_ system are shown with several slab geotherms^31^ in Fig. 3. The slab geotherms in Fig. 3a are models based on the geological observations^32^ and mineralogical hypothesis^31^ in the present Earth. The super-deep diamonds form Juina kimberlite were considered to be related to Gondwana subduction and formed at 150 Ma^5^. Therefore, the colder geotherms like the modern Earth may be useful to be compared with the MgCO_3_-SiO_2_ reactions. The reactions in this system provide important information for understanding the stability limits of MgCO_3_ in deep subduction.

The reaction between MgCO_3_ and SiO_2_ may not occur down to the top of the lower mantle but can occur at depths from 1,000 to 2,000 km in various subducting slabs. In “hot” and “cold” slabs^31^, which are comparable to Mexico and the various Pacific subduction paths respectively^32^, magnesite can react with stishovite to form bridgmanite and CO_2_ at depths of around 1,700 km, and subsequently CO_2_ can decompose to diamond and O_2_ at the greater depths. Therefore, super-deep diamond could be formed at depths greater than 1,700 km in cold slabs.

The path of “very cold” slabs^31^ such as Tonga subduction^32^ may pass through the ‘bottleneck’ region in the phase diagram at ~80 GPa corresponding to the depth near the MgCO_3_ magnesite-phase II boundary (~1,900 km). MgCO_3_ in such slabs may descend to the base of the lower mantle because the stability field of MgSiO_3_ + diamond + O_2_ is relatively narrow or absent in the pressure-temperature path of very cold slabs (Fig. 3a). If MgCO_3_ is able to survive beyond 1,900-km depth, MgCO_3_ phase II will be formed and subsequently decompose to MgSiO_3_ post-perovskite phase + diamond + O_2_ by a reaction with seifertite at the base of the lower mantle due to heating from the outer core.

Slabs in the early Earth, Archean/Proterozoic age, might descend into the hotter mantle than the modern adiabat^33^. The hotter subduction could restrict the MgCO_3_ subduction into the deep lower mantle because of the reaction (1) in the shallower mantle (Fig. 3). In this case, CO_2_ might be a significant carbon carrier in the subducted slabs although its stability has been still controversial under high pressure and high temperature conditions^28^,^30^. If early slab temperatures had been higher than the modern mantle adiabat^33^, the formation of CO_2_ fluid^28^ or melting in the MgCO_3_-SiO_2_ system^26^ would be expected down to the mantle transition zone. These phenomena might prevent the subduction of carbon-bearing phases into the lower mantle. This might be the reason why the reports of the old super-deep diamonds are absent.

The reaction in the “very cold” slab may play an important role in formation of super-deep diamond in the deep lower mantle. Reference 5 summarized the mineral inclusions in diamond, and estimated the formation depths of diamonds in the mantle. Some diamonds from São Luiz, Juina province, Brazil have inclusions of orthopyroxene which are a psuedomorph of bridgmanite and TAPP (tetragonal almandine-pyrope phase) suggesting the top of the lower mantle, 660–750 km depths. They also suggested that some diamonds with inclusions of aluminous bridgmanite pseudomorph from the same locality, Juina, Brazil, might be originated at the depths greater than 750 km, although the exact depth limit was not estimated. On the other hand, Diamonds containing the iron-rich (Mg,Fe)O inclusions^6^,^8^ were considered to be originated from the deep lower mantle (1,700–2,900 km) because of iron enrichment by the spin transition at depths greater than about 1,700 km^34^,^35^,^36^, or by the phase transition from bridgmanite to post perovskite at around 2,700-km depth^37^,^38^,^39^,^40^,^41^, although it is a debated matter whether iron-rich (Mg,Fe)O inclusions are the signature of the bottom of the lower mantle or not.

The mechanism of diamond formation discovered here may explain the origin of super-deep diamond from the deep lower mantle such as those reported from the limited locality in Brazil^5^,^6^,^8^. Although we did not consider the effects of *f*O_2_^42^,^43^ in the reaction (3) because of the technical difficulty for controlling *f*O_2_ in the DAC experiments, we can expect the above reaction in the presence of iron in the average lower mantle with the *f*O_2_ condition below the iron-wüstite buffer^44^. When metallic iron exists in the system, iron oxide can be formed by the reaction with O_2_ in the run products. The role of iron is also important in formation of high-pressure polymorphs of carbonate^18^,^45^,^46^. The latest studies reported that the iron-magnesium carbonate transforms into the several new phases: Fe_4_(CO_4_)_3_ phase having unknown structure^18^, orthorhombic (Mg,Fe)CO_3_ phase II^45^, or the unquenchable phases with unexpected stoichiometry (Mg_2_Fe_2_(C_4_O_13_) + Fe_13_O_19_) that coexist under the lower mantle^46^. Therefore, we may need to modify the present phase relation further in the iron bearing system. However, we can consider that the reaction of diamond formation could also occur in the iron-bearing system since iron oxides and/or FeO-bearing bridgmanite and ferropericlase can be formed in deep subducted slabs.

Released oxygen will oxidize the ferrous iron in mantle minerals or the metallic iron penetrated from the outer core at the core-mantle boundary^28^,^47^. The oxidation of metallic iron can generate FeO. As a result, the iron-rich magnesiowüstite of (Mg,Fe)O^6^,^8^ would be formed as inclusions in the deepest diamonds, although it is not a single process for generation of FeO enrichment in magnesiowustite as inclusions in diamond and other processes of the redox change in the shallower depths could also have generated similar iron enrichment. Future studies are needed to elucidate the ultra-deep iron-carbon redox coupling processes and their influence on formation of super-deep diamond.

## Methods

A natural single crystal of magnesite (Bahia, Brazil, Mg_0.994_Ca_0.003_Fe_0.003_CO_3_) and a reagent quartz (reagent grade, Woko) were ground to fine powders in an agate mortar for 1 hour. The powders were mixed 1:1 by mole fraction and ground in an agate mortar for 30 min to homogenize the mixture. High-pressure and high-temperature experiments were conducted using a double sided LHDAC. The culet diameters of the diamond anvils were between 100 and 350 μm. The sample chamber drilled in rhenium or tungsten gaskets ranges from 30 to 100 μm in diameter and from 40 to 80 μm in the thickness depending on the culet sizes. NaCl or SiO_2_ glass was used as the pressure medium and thermal insulator.

Platinum was included in the sample chamber as a laser absorber. We used three sample configurations to obtain stable temperatures at high pressures: Run Ch1-3-layer-chamber, Run Ch2-5-layer-chamber and Run Ch3-Pt/W-doughnut-chamber (Supplementary Table 1). In Run Ch1, 5 wt. % platinum powder (platinum black, purity 99.9%, Mitsuwa Chemicals) was mixed with magnesite and quartz starting material (Ch1) and this was sandwiched by a pressure medium. On the other hand, in Run Ch2, the magnesite and quartz mixture was sandwiched by platinum foils (purity 99.9%, Nilaco). Run Ch2 aimed to reduce the temperature gradient at the heating spot during double-sided laser heating.

The samples were heated by double-sided fiber lasers (SPI LASER) of wavelength 1.090 μm at Tohoku University in Sendai, Japan and 1.070 μm at BL10XU of SPring-8 in Hyogo, Japan. The heating duration was 10–120 minutes. Temperature was determined by fitting the emission spectra from the heated samples to the gray-body radiation formula.

*In situ* synchrotron X-ray diffraction (XRD) was conducted to identify the experimental products. We acquired the XRD patterns of the samples at high pressure up to 152 GPa in the temperature range from 300 K to 3,100 K at BL10XU of SPring-8. The collimator was 20 μm in diameter and the typical wavelength of X-ray was 0.41414(9) Å. An imaging plate (Rigaku, R-AXIS IV^++^) and a charge coupled device (Bruker, AXS SMART APEX) detectors were used for acquiring the XRD patterns. The pressures of *in situ* experiments were determined based on the equation of state of Pt^48^. Thermal pressure was estimated based on Mie–Grüneisen–Debye model^49^,^50^. In Runs Ch3-1 and Ch2-5, the sample was heated at Tohoku University and observed after temperature quenching at high pressure using XRD at BL10XU of SPring-8. In Ch3-1, the sample was recovered to the ambient condition at Tohoku University after the temperature quenching and pressure determination using ruby fluorescence method^51^. Since thermal pressure could not be estimated in Runs Ch3-1 and Ch2-5, we considered the pressure errors up to ±10 GPa for pressures at high temperature during laser heating. This is almost equivalent to the maximum pressure increase by laser heating in the *in situ* XRD experiments.

The high-pressure phase transition of MgCO_3_ was confirmed in two series of runs, Ms-1 and Ms-2 in addition to the MgCO_3_-SiO_2_ system (Supplementary Table 2). These runs were conducted using a membrane-type diamond anvil cell. The natural magnesite and Pt powder were sandwiched by the same magnesite in the sample chamber. Pt powder was used as a pressure maker and laser absorber. Magnesite was compressed to about 100 GPa, and then pre-heated to temperature of less than 1,500 K using CO_2_ laser at Tohoku University in order to crystallize the compressed sample in Run Ms-1. We conducted laser heating at 2,500–3,000 K for 5–20 min in order to obtain the obvious XRD patterns of the MgCO_3_ high-pressure phase using SPI fiber laser in two series of runs, Ms-1 and Ms-2. XRD patterns were acquired at high pressure and 300 K after quenching.

Temperatures in some experiments increased suddenly to above 3,000 K in the Runs Ch1-1, Ch1-2, Ch1-3 and Ch 2-1 using platinum as a laser absorber (Supplementary Table 1). This phenomenon may correspond to the ‘temperature jump’ reported by ref. 13. The sudden temperature increase was also observed at high pressures and above 2,000 K when a Pt powder (Run Ch1) and Pt foil (Run Ch2) was used as the laser absorber. These results indicate that large temperature fluctuation may be caused by the volume change following reactions, phase transitions and/or melting of the sample.

## Additional Information

**How to cite this article**: Maeda, F. *et al*. Diamond formation in the deep lower mantle: a high-pressure reaction of MgCO_3_ and SiO_2_. *Sci. Rep.* **7**, 40602; doi: 10.1038/srep40602 (2017).

**Publisher's note:** Springer Nature remains neutral with regard to jurisdictional claims in published maps and institutional affiliations.

## Supplementary Material

Supplementary Information
