# Supplementary material for: Diamond formation in the deep lower mantle: a high-pressure reaction of MgCO3 and SiO2
Source: Sci Rep. 2017 Jan 13;7:40602. doi: 10.1038/srep40602 (PMC5233982; doi:10.1038/srep40602)
Supplement: Supplementary Information [file srep40602-s1.pdf]

Supplementary Information

Diamond formation in the deep lower mantle: a high-pressure reaction of $MgCO_3$ and SiO_2

Fumiya Maeda[1], Eiji Ohtani[1,2], Seiji Kamada[1,3], Tatsuya Sakamaki[1], Naohisa Hirao[4], Yasuo Ohishi[4]

[1] *Department of Earth Science, Graduate School of Science, Tohoku University, Sendai, 980-8578, Japan*

[2] *V.S. Sobolev Institute of Geology and Mineralogy, SB RAS, Novosibirsk, 630090, Russia*

[3] *Frontier Research Institute for Interdisciplinary Sciences, Tohoku University, Sendai, 980-8578, Japan*

[4] *Japan Synchrotron Radiation Research Institute, Sayo, Hyogo 679-5198, Japan*

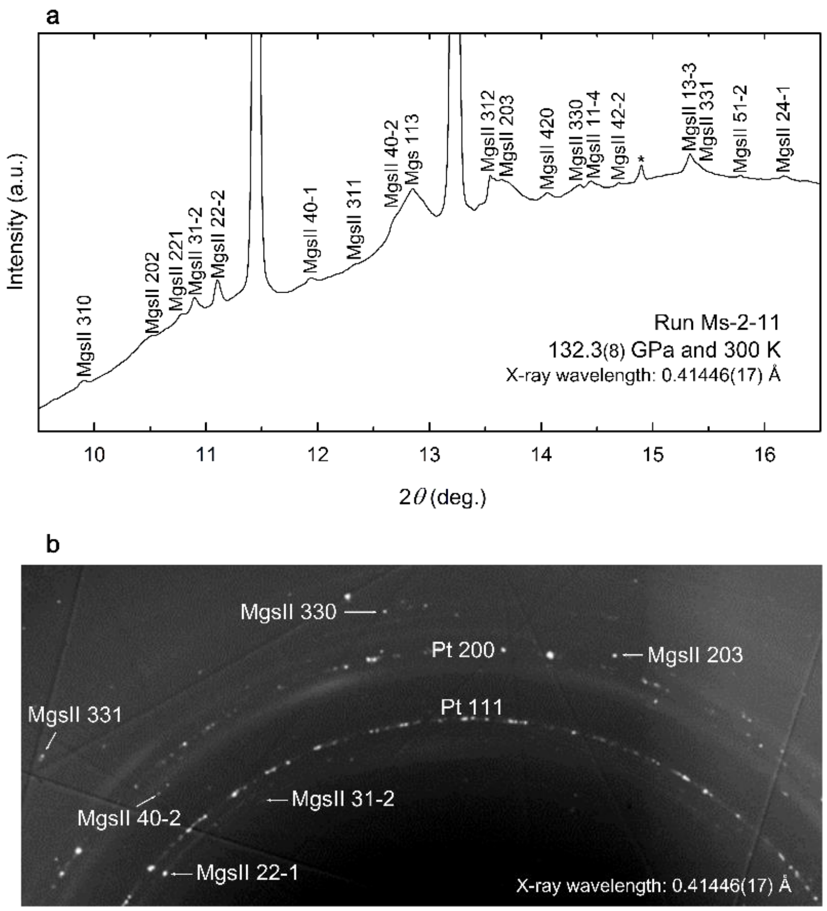

Supplementary Figure S1. XRD patterns of $MgCO_3$ phase II at 132.3 (8) GPa and 300 K. Representative XRD patterns of $MgCO_3$ high-pressure polymorph, phase II. (a) The in situ XRD pattern obtained at 132.3 (8) GPa and 300 K. The diffraction peaks were indexed based on a monoclinic symmetry (space group C2/m) and reported crystal parameters[1]. The $MgCO_3$ sample was heated at 146.4 (32) GPa and 2,440 (240) K for 5 minutes before acquiring this pattern at 132.3 (8) GPa and 300 K. (b) The 2D XRD image obtained at 132.3 (8) GPa and 300 K. Mgs and MgsII represent magnesite and $MgCO_3$ phase II, respectively.

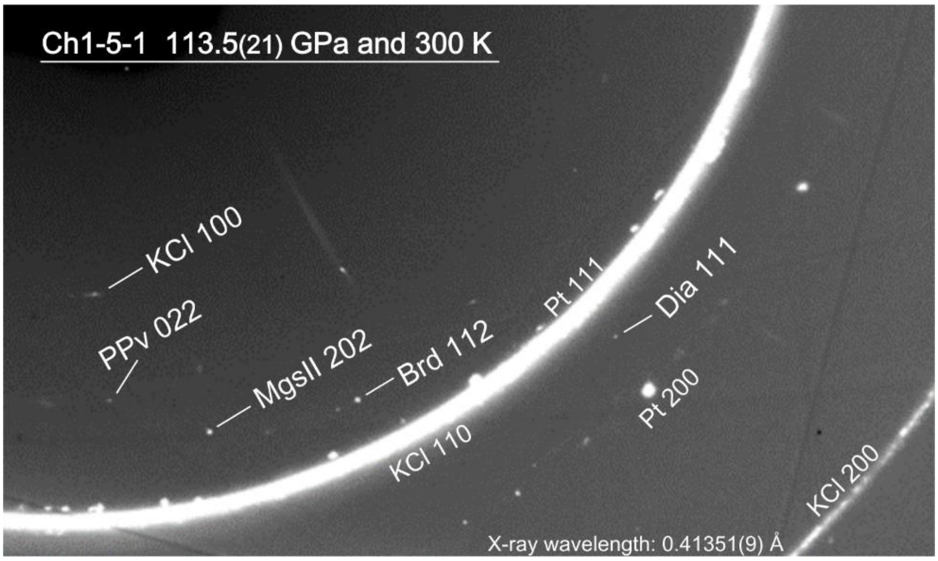

Supplementary Figure S2. XRD pattern of the $MgCO_3$-SiO_2 sample quenched from 131.1 (32) GPa and 2,540 (370) K. The *in situ* XRD pattern obtained from 113.5 (21) GPa and 300 K after laser heating at 131.1 (32) GPa and 2,540 (370) K. The abbreviations represent as follow: MgsII: $MgCO_3$ phase II, PPv: $MgSiO_3$ post-perovskite phase, Brd: bridgmanite, Dia: diamond, Pt: platinum, KCl: potassium chloride (pressure medium).

Supplementary Table S1. The experimental conditions and run products.

Run No.	Pressure (GPa)	Temperature (K)	Observed phases	Analysis	Sample chamber (Pressure medium)	duration (min)
Ch1-1	82.2 (10)	1,700 (190)	Mgs + CS + Brd	$XRD_{HPT;AC}$	Ch1 (NaCl)	60
	83.1 (5)	1,780 (210)	Mgs + MgsII + CS + Brd + Dia + CO_2-VI	$XRD_{HPT;AC}$	Ch1 (NaCl)	60
	~85	>3,000^{TJ}	MgsII + St + Brd + Dia (+ CO_2-VI?)	$XRD_{HPT;AC}$	Ch1 (NaCl)	45
Ch1-2	67.5 (4)	1,980 (160)	Mgs + St + Brd	$XRD_{HPT;AC}$	Ch1 (SiO_2 glass)	60
	64.4 (5)	2,290 (160)	Mgs + St + Brd	XRD_{HPT}	Ch1 (SiO_2 glass)	25
	~70	>3,000^{TJ}	Mgs + St + Brd	$XRD_{HPT;AC}$	Ch1 (SiO_2 glass)	60
Ch1-3	26.7 (6)	1,490 (110)	Mgs + St	XRD_{HPT}	Ch1 (NaCl)	60
	~30	>3,000^{TJ}	Mgs + St + Brd	XRD_{HPT}	Ch1 (NaCl)	20
Ch1-4	138.2 (66)	1,470 (30)	MgsII + Se	XRD_{HPT}	Ch1 (SiO_2 glass)	60
	133.1 (51)	2,140 (240)	MgsII + Se	XRD_{HPT}	Ch1 (SiO_2 glass)	60
Ch1-5	131.1 (32)	2,540 (370)	MgsII + Se + Brd + PPv + Dia	XRD_{HPT}	Ch1 (KCl)	60
Ch2-1	72.5 (3)	1,700 (140)	Mgs + St(CS)	XRD_{HPT}	Ch2 (SiO_2 glass)	120
	73.6 (3)	1,890 (170)	Mgs + St(CS)	XRD_{HPT}	Ch2 (SiO_2 glass)	30
	~70	>3,000^{TJ}	Mgs + St(CS) + Brd	XRD_{HPT}	Ch2 (SiO_2 glass)	30
Ch2-2	29.9 (6)	1,550 (90)	Mgs + St	$XRD_{HPT;AC}$	Ch2 (SiO_2 glass)	60
	34.4 (14)	1,990 (180)	Mgs + St	$XRD_{HPT;AC}$	Ch2 (SiO_2 glass)	125
	40.2 (15)	2,110 (190)	Mgs + St	$XRD_{HPT;AC}$	Ch2 (SiO_2 glass)	60
	36.3 (27)	2,290 (310)	Mgs + St + Brd	$XRD_{HPT;AC}$	Ch2 (SiO_2 glass)	70
Ch2-3	65.2 (7)	1,770 (70)	Mgs + St(CS)	XRD_{HPT}	Ch2 (SiO_2 glass)	60
	71.1 (11)	2,030 (90)	Mgs + St(CS) + Brd	XRD_{HPT}	Ch2 (SiO_2 glass)	95
	70.6 (13)	2,170 (160)	Mgs + St(CS) + Brd	XRD_{HPT}	Ch2 (SiO_2 glass)	60
	~70	>3,000 ^{TJ}	Mgs + St(CS) + Dia	XRD_{HPT}	Ch2 (SiO_2 glass)	15
Ch3-1	78.4 (100)	2,200 (280)	Mgs + St(CS) + Brd + Dia	XRD_{AC}	Ch3 (NaCl)	60
Ch3-2	103.3 (18)	2,140 (210)	Mgs + MgsII + CS + Brd + Dia (+ hpMgs?)	XRD_{HPT}	Ch3 (SiO_2 glass)	80
Ch3-3	145.5 (7)	2,000 (80)	MgsII + Se + PPv + Dia	$XRD_{HPT;AC}$	Ch3 (SiO_2 glass)	165
	145.5 (31)	2,700 (360)	MgsII + Se + PPv + Dia	$XRD_{HPT;AC}$	Ch3 (SiO_2 glass)	70
	152.5 (34)	3,110 (320)	MgsII + Se + PPv + Dia	$XRD_{HPT;AC}$	Ch3 (SiO_2 glass)	60
Ch2-4	92.9 (13)	1,880 (150)	Mgs + CS + Brd + Dia (+ hpMgs?)	XRD_{HPT}	Ch2 (SiO_2 glass)	60
	118.9 (21)	2,030 (90)	Mgs + CS (+ hpMgs?)	XRD_{HPT}	Ch2 (SiO_2 glass)	15
Ch2-5	85.4 (99)	1,440 (80)	Mgs + CS	$XRD_{HP;AC}$	Ch2 (SiO_2 glass)	90

The subscripts of XRD shows the condition where the XRD patterns were acquired: HPT: at high-pressure and high-temperature; AC; under the ambient condition. The subscript of TJ shows the temperature jump was observed on heating. The values in the parenthesis after pressure and temperature shows uncertainty. The abbreviations represent as follow: Mgs: magnesite, St: stishovite, CS: $CaCl_2$-type SiO_2 phase, Brd: bridgmanite, Dia: diamond, MgsII: $MgCO_3$ phase II, PPv: $MgSiO_3$ post-perovskite phase. hpMgs: high-pressure $MgCO_3$ phase: Two (103.3 GPa and 2,140 K) or four (92.9 GPa and 1,880 K) weak diffraction peaks perhaps from other high-pressure phases of $MgCO_3$, for instance, P-1 phase[4], were observed at high pressure and temperature. We need further studies to characterize the phases.

Supplementary Table S2. Crystal parameters of $MgCO_3$ high-pressure polymorphs.

	P (GPa)	*a* (Å)	*b* (Å)	*c* (Å)	α (deg.)	β (deg.)	γ (deg.)	V (Å^3)
<u>This study</u>	Phase II: monoclinic (Space group: C2/m)							
(Run No.)	T = 300 K							
Ms-1-1	84.9 (6)	8.187 (8)	6.613 (3)	6.992 (7)	90	104.28 (5)	90	366.9 (7)
Ms-1-2	87.1 (8)	8.180 (8)	6.608 (3)	6.991 (7)	90	104.14 (6)	90	366.4 (7)
Ms-1-3	89.4 (8)	8.157 (11)	6.590 (6)	6.973 (8)	90	104.53 (6)	90	362.8 (10)
Ms-1-4	93.9 (6)	8.137 (11)	6.584 (6)	6.952 (9)	90	104.56 (7)	90	360.5 (10)
Ms-2-1	97.3 (11)	8.156 (5)	6.539 (3)	6.930 (5)	90	103.99 (4)	90	358.6 (5)
Ms-2-2	102.1 (4)	8.138 (5)	6.526 (4)	6.925 (5)	90	103.93 (3)	90	357.0 (6)
Ms-2-3	107.0 (3)	8.113 (4)	6.512 (2)	6.897 (5)	90	103.84 (3)	90	353.8 (5)
Ms-2-4	111.4 (8)	8.093 (6)	6.492 (3)	6.869 (5)	90	103.80 (4)	90	350.5 (5)
Ms-2-5	117.7 (8)	8.063 (4)	6.457 (2)	6.854 (4)	90	103.85 (3)	90	346.5 (4)
Ms-2-6	121.3 (5)	8.047 (5)	6.459 (4)	6.847 (5)	90	103.81 (3)	90	345.6 (5)
Ms-2-7	122.7 (8)	8.031 (5)	6.444 (2)	6.833 (5)	90	103.84 (3)	90	343.3 (5)
Ms-2-8	124.5 (6)	8.027 (6)	6.439 (3)	6.830 (5)	90	103.87 (4)	90	342.7 (5)
Ms-2-9	127.1 (7)	8.013 (5)	6.431 (3)	6.817 (6)	90	103.82 (4)	90	341.1 (6)
Ms-2-10	129.2 (6)	8.014 (5)	6.424 (3)	6.805 (6)	90	103.78 (4)	90	340.3 (6)
Ms-2-11	132.3 (8)	7.996 (9)	6.421 (4)	6.799 (8)	90	103.82 (6)	90	338.9 (8)
Ch3-3	126.2 (16)	7.904 (30)	6.464 (20)	6.839 (13)	90	103.76 (9)	90	339.3 (26)
<u>Oganov *et al.* (2008)[1]</u>		82-138 GPa: Phase II (C2/m); 138-160 GPa: Phase III ($P2_1$)						
(Calculation: T = 300 K)								
Phase II (C2/m)	120	8.0945	6.4881	6.8795	90	103.98	90	350.60
Phase III ($P2_1$)	120	4.5338	7.7918	5.0864	90	104.54	90	173.93
<u>Boulard *et al.* (2011)[2]</u>		Phase II ($P2_1/c$)						
(Experiment: T = 2,400 K)								
Phase II ($P2_1/c$)	85	8.37	6.41	6.82	90	104.57	90	351.7
<u>Pickard and Needs (2015)[4]</u>		85-101 GPa: P-1 (post magnesite); ~144 GPa: $P2_12_12_1$ (post phase II)						
(Calculation: T = 300 K)								
P-1	100	5.211	5.238	7.268	70.030	81.904	78.272	182.01
$P2_12_12_1$	200	9.436	7.505	4.355	90	90	90	299.27

Supplementary Table S3. Crystal parameters of magnesite in the $MgCO_3$-SiO_2 system.

Run No.	Pressure (GPa)	Temperature (K)	*a* (Å)	*c* (Å)	*V* (Å^3)
Ch1-1	82.2 (10)	1,700 (190)	(unable to fit)		
	83.1 (5)	1,780 (210)	(unable to fit)		
Ch1-2	67.5 (4)	1,980 (160)	4.3346 (5)	12.989 (5)	211.4 (2)
	64.4 (5)	2,290 (160)	4.3590 (11)	13.076 (6)	215.2 (3)
	~70	>3,000^{TJ}	4.3573 (8)	13.084 (4)	215.1 (2)
Ch1-3	26.7 (6)	1,490 (110)	4.4880 (18)	14.057 (24)	245.2 (10)
	~30	>3,000^{TJ}	4.4624 (12)	13.912 (6)	239.9 (2)
Ch2-1	72.5 (3)	1,700 (140)	(unable to fit)		
	73.6 (3)	1,890 (170)	4.3302 (7)	12.931 (10)	210.0 (4)
	~70	>3,000^{TJ}	4.3310 (14)	12.976 (15)	210.8 (6)
Ch2-2	29.9 (6)	1,550 (90)	4.4665 (10)	13.902 (6)	240.2 (2)
	34.4 (14)	1,990 (180)	4.4507 (8)	13.841 (4)	237.5 (2)
	40.2 (15)	2,110 (190)	4.4345 (12)	13.714 (13)	233.5 (5)
	36.3 (27)	2,290 (310)	4.4581 (14)	13.890 (14)	239.1 (6)
Ch2-3	65.2 (7)	1,770 (70)	4.3389 (7)	13.024 (4)	212.5 (2)
	71.1 (11)	2,030 (90)	4.3248 (18)	12.870 (9)	208.5 (4)
	70.6 (13)	2,170 (160)	4.3288 (26)	12.967 (15)	210.4 (6)
	~70	>3,000^{TJ}	4.3274 (25)	12.838 (16)	208.2 (6)
Ch3-2	103.3 (18)	2,140 (210)	(unable to fit)		
Ch2-4	92.9 (13)	1,880 (150)	4.2610 (54)	12.531 (41)	197.0 (16)
	118.9 (21)	2,030 (90)	4.2638 (25)	12.339 (15)	194.3 (6)
Ch2-5	85.4 (99)	300	4.2799 (31)	12.588 (28)	199.7 (11)

"(unable to fit)" shows that we were unable to estimate the lattice parameters in a few XRD patterns because the number and intensity of peaks were not enough to be fitted.

Supplementary References

1. Oganov, A. R. *et al*. Novel high-pressure structures of $MgCO_3$, $CaCO_3$ and CO_2 and their role in Earth's lower mantle. *Earth Planet. Sci. Lett.* **273**, 38-47 (2008)
2. Boulard, E. *et al*. New host for carbon in the deep Earth. *Proc. Natl Acad. Sci. USA* **108**, 5184-5187 (2011)
3. Pickard, C. J. & Needs, R. J. Structures and stability of calcium and magnesium carbonates at mantle pressures. *Phys. Rev. B* **91**, 104101 (2015)
